# Epidemiology of Invasive Fungal Diseases: A 10-Year Experience in a Tertiary Pediatric Hematology–Oncology Department in Greece

**DOI:** 10.3390/jof10070498

**Published:** 2024-07-18

**Authors:** Eleni Moraitaki, Ioannis Kyriakidis, Iordanis Pelagiadis, Nikolaos Katzilakis, Maria Stratigaki, Georgios Chamilos, Athanasios Tragiannidis, Eftichia Stiakaki

**Affiliations:** 1 Department of Pediatric Hematology-Oncology & Autologous Hematopoietic Stem Cell Transplantation Unit, University Hospital of Heraklion & Laboratory of Blood Diseases and Childhood Cancer Biology, School of Medicine, University of Crete, 71003 Heraklion, Greece; ra8820027@yahoo.com (E.M.); kyriakidis@med.uoc.gr (I.K.); ipelagiadis@icloud.com (I.P.); katzilaher@yahoo.gr (N.K.); mstratigaki@hotmail.com (M.S.); 2 MSc Program “Hematology-Oncology of Childhood and Adolescence”, School of Medicine, University of Crete, 71003 Heraklion, Greece; 3 Clinical Microbiology and Microbial Pathogenesis Department, School of Medicine, University of Crete, 71003 Heraklion, Greece; hamilos@uoc.gr; 4 Pediatric & Adolescent Hematology Oncology Unit, 2nd Pediatric Department, AHEPA Hospital, Faculty of Health Sciences, Aristotle University of Thessaloniki, 54636 Thessaloniki, Greece; atragian@auth.gr

**Keywords:** invasive fungal diseases, cancer, child, adolescent, epidemiology, risk factors, mycoses, aspergillosis, *Candida*, prophylaxis

## Abstract

Although advances in the management of pediatric neoplasms have profoundly improved infectious disease outcomes, invasive fungal diseases (IFDs) remain a major cause of morbidity and mortality in children and adolescents with high-risk hematological malignancies. A retrospective study was conducted in the Pediatric Hematology–Oncology Department of the University General Hospital of Heraklion for 2013–2022 to estimate the prevalence and describe the clinical and epidemiological characteristics of IFDs for pediatric and adolescent patients with neoplasia. Demographic, clinical, and laboratory parameters were analyzed to identify risk factors for the development of IFD. The overall prevalence of IFDs was estimated to be 7.8% (12/154 patients) throughout the study. The mean age at IFD diagnosis was 9.8 years (SD 6.4 years). The most common IFD was possible/probable invasive pulmonary aspergillosis (IPA; in ≈50%), followed by candidemia/invasive candidiasis (in 44%). *Candida parapsilosis* was the most prevalent *Candida* species (4/6 events). Of interest, the majority (75%) of IFDs were breakthrough infections. Patients with increased risk for IFDs were those who were colonized by fungi in sites other than the oral cavity, hospitalized in the intensive care unit for >7 days, received >7 different antimicrobials in the last 3 months, or had severe neutropenia for >44 days. Two children out of a total of 12 with IFD died due to refractory disease or relapse (16.7%). More detailed and prospective epidemiological studies on fungal infections in pediatric patients with hematological or solid neoplasms can contribute to the optimization of prevention and treatment.

## 1. Introduction

Children with malignancy are predisposed to invasive fungal diseases (IFD) due to severe acquired immunodeficiency as a result of the underlying disease, intensive chemotherapy, steroid treatment, or Hematopoietic Stem Cell Transplantation (HSCT) [1,2]. The incidence of IFDs in these children depends upon geographical region, the underlying malignant disease, the intensity of the chemotherapy treatment, and the patient’s characteristics [3]. Recent studies estimate the prevalence of IFDs at 5.9% among pediatric patients with cancer, varying between 2% and 28% in other studies in the field [4,5].

Diagnosis of IFDs is challenging due to the invasiveness of the procedure, as in the case of lung biopsy for pulmonary mycosis or due to the low yield of positive cultures, especially when the patient is under antifungal prophylaxis. Knowledge of local epidemiology and current international guidelines are crucial in deciding appropriate prophylaxis and empirical and pre-emptive treatment of IFDs. A personalized risk assessment is also warranted for the proper use of antifungals to avoid overtreatment and shift the epidemiological profile to more resistant and emerging fungi, such as those belonging to the *Mucorales* family [6,7,8].

Prevention, early identification, and prompt treatment are key elements affecting pediatric Hematology–Oncology patients’ IFDs course and outcome. Consensus definitions of IFDs have been recently revised and updated by the European Organisation for Research and Treatment of Cancer and Mycoses Study Group Education and Research Consortium (EORTC/MSGERC). Still, their validity and application in the pediatric population require careful assessment [9]. In the same context, the eighth European Conference on Infections in Leukaemia (ECIL-8) has updated the guidelines for the diagnosis, prevention, and treatment of IFDs in pediatric patients with cancer or post-HSCT [10]. Guidelines on the diagnosis of invasive aspergillosis in the pediatric population have also been issued by the European Society for Clinical Microbiology and Infectious Diseases (ESCMID) and the European Confederation of Medical Mycology (ECMM) [11]. All these publications include guidance on microbiologic, histopathologic, radiologic, and clinical identification of IFDs in neonates, children, and adolescents with malignant diseases.

The current study intends to investigate the occurrence and epidemiology of IFDs in a single Pediatric Hematology–Oncology Department, identify predisposing factors, and describe the clinical features and outcomes in the respective cases.

## 2. Patients and Methods

A retrospective single-center study included all children and adolescents (up to 19 years old) diagnosed and treated with neoplasia in the Pediatric Hematology–Oncology Department in Heraklion, Crete. The medical records of all patients corresponding to the period from 2013 to 2022 were registered, and relevant data were collected. The EORTC/MSGERC consensus host factors for IFDs were recorded: a recent history of prolonged neutropenia (<500 neutrophils/μL for >10 days), hematologic or not malignancy, allogeneic HSCT or solid organ transplant, prolonged use of corticosteroids (≥0.3 mg/kg for ≥three weeks in the past 60 days), treatment with B-cell or T-cell immunosuppressants, severe hereditary immunodeficiency, and graft-versus-host disease (GVHD) [9]. Demographic data, clinical characteristics, diagnostic exams, treatment protocol, comorbidities, intensive care unit (ICU) hospitalization, neutrophils, lymphocytes, monocytes count, the duration of severe neutropenia, lymphopenia and monocytopenia, number of antimicrobials’ courses administered during the last three months before the onset of the IFD (including both antibiotics and antifungals), the antifungal prophylaxis for at least seven days before the onset of symptoms due to IFD, the clinical, laboratory and radiological characteristics of IFDs, and finally the specific treatment that followed were also recorded. The effect of each categorical variable on the development of IFD was investigated utilizing the chi-square and Fisher’s exact tests. Respectively, the Mann–Whitney U and Wilcoxon tests were used for the continuous variables. The value of *p* ≤ 0.05 was considered statistically significant. The statistical analysis was performed using the IBM SPSS Statistics V22 package (IBM Corp., Armonk, NY, USA). The prevalence of IFDs was calculated by dividing the number of cases by the total number of patients at risk. Definitions of IFDs (proven, probable, and possible) in this study adhered to EORTC criteria [9]. Breakthrough IFD was defined as any invasive fungal infection occurring during exposure to an antifungal drug, according to the respective MSGERC position paper [12]. The clinical practice guideline by the International Pediatric Fever and Neutropenia Guideline Panel was utilized for febrile neutropenia [13].

The study was conducted in accordance with the Declaration of Helsinki, approved by the Institutional Review Board of the University General Hospital of Heraklion, and carried out according to its regulations (decision 352/27 February 2024; Scientific Council 2088/5 February 2024).

## 3. Results

In total, 154 patients’ medical records were reviewed. Of the patients aged from 32 days to 20 years, 57% were male and 43% were female. The mean age at diagnosis was 8.7 years and the most common malignancy was acute lymphoblastic leukemia (ALL; 27%), followed by lymphomas (23%), central nervous system tumors (CNS; 19%), sarcomas (9%), neuroblastoma (8%), acute myeloid leukemia (AML; 6%), kidney tumors (4%), chronic myeloid leukemia (2%), and other neoplasia (2%). Patients’ residence at a distance more than 100 km from the Department (28.8%) was not associated with IFD occurrence.

The crude prevalence of IFDs was 7.8% (12/154 patients). Sixteen IFDs were recorded in 12 patients, of which eight were proven (50%), one was probable (6.3%), and seven were possible IFDs (43.7%). The IFD rates have increased by 185% during the five-year period 2018–2022 compared to 2013–2017 (10% vs. 3.5%). The mean age at IFD diagnosis was 9.8 years, and the most common IFD was possible/probable invasive pulmonary aspergillosis (IPA) in half of these cases, followed by fungemia (37.5%), *Candida* cellulitis (6.3%), and *Pneumocystis jirovecii* pneumonia (PJP; 6.3%). No sex predilection was noted (58% males vs. 42% females), and age spanned from 7 months to 19 years old (with half of patients aged >12 years old).

One patient experienced three IFDs and was recorded three times in the datasheet; another one had two IFDs, while one patient had an IFD relapse. The two latter patients were recorded twice for the statistical analysis. Breakthrough infections accounted for 87.5% of IFDs. Most patients have been receiving micafungin as primary antifungal prophylaxis (42%), which was the most common prophylaxis administered across all chemotherapy regimens in this study, followed by liposomal amphotericin B (LAMB; 17%), and caspofungin, fluconazole, voriconazole, and pentamidine (6.7% each). Two patients have been not eligible for prophylactic antifungal treatment (20%).

**Figure 1:**
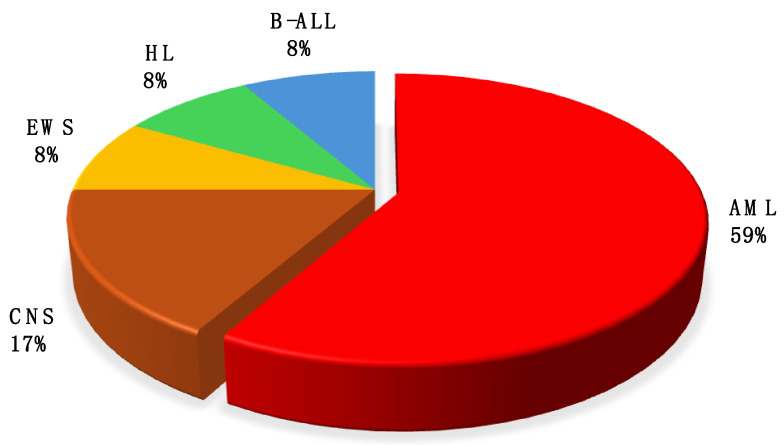
Underlying neoplasia in children who developed IFD. AML = Acute Myeloid Leukemia; B-ALL = B-Cell Acute Lymphoblastic Leukemia; CNS = Brain tumors; HL = Hodgkin Lymphoma; EWS = Ewing Sarcoma.

The underlying malignancies of the patients with IFD are demonstrated in Figure 1. AML M5, according to the FAB classification, was the most common underlying neoplasia, which was also associated with significantly higher mortality rates, as both patients who succumbed to an IFD were diagnosed with AML M5 and were over 12 years old. Interestingly, seven out of 10 AML cases in total during the observation period developed an IFD. Contrarily, only one patient out of 39 recorded ALL patients manifested an IFD (2.6%), and he was being administered with LAMB, while all other ALL patients were treated with prophylactic micafungin. All AML patients received prophylaxis with micafungin concurrent with or followed by LAMB.

The association of clinical and laboratory parameters with the IFD occurrence is shown in Table 1. The development of an IFD was associated with an ICU hospitalization for more than seven days (31% vs. 4%; *p* = 0.017), the administration of more than seven different antimicrobial courses during the last three months (50% vs. 16%; *p* = 0.01), the history of severe neutropenia for more than 44 days (38% vs. 17%; = 0.011), or the colonization by fungi in sites other than the oral cavity, and specifically the throat, the urinary bladder, and indwelling catheters (31% vs. 6%; *p* = 0.007). The administration of prednisolone equivalent ≥ 0.3 mg/kg for ≥3 weeks also seems to confer a significantly increased risk for an IFD. The absolute number of lymphocytes or monocytes did not seem to be an independent risk factor for IFD. An axillary body temperature greater than 38.8 °C for more than 12 days and a C-reactive protein (CRP) greater than 10 mg/dL were associated with IFDs (50% vs. 29% with *p* = 0.019; 44% vs. 6% with *p* < 0.001; and 38% vs. 22% with *p* = 0.011, respectively). According to the statistical analysis, the prophylactic administration of antifungals and granulocyte colony-stimulating factor (GCSF) were not associated with the development of an IFD. Conversely, the administration of IVIG seems to be significantly associated with the development of an IFD, but this finding can be attributed to the increased rates of IVIG administration when an IFD is suspected. HSCT and therapy with immunomodulatory agents, such as monoclonal antibodies or small molecule inhibitors, have not been proven as independent risk factors for IFD development. The prevalence of comorbidities, such as endocrine disorders, genetic syndromes, kidney disease, thrombosis, and cardiovascular disorders, did not differ significantly between cases with IFD and the rest of the cohort. In addition, the rate of relapsed or refractory (r/r) underlying disease in this retrospective cohort study was calculated at 13%, and even though r/r cases were more frequent among patients with IFD, no significant effect was noted.

Investigations among IFD cases revealed that CRP was significantly higher in patients who succumbed to the IFD compared to those who recovered (21 ± 3.7 vs. 9.7 ± 6.1; *p* = 0.017). No other parameter was correlated significantly with IFD-related deceased cases. Of note, both patients with IFD who experienced a relapse of the underlying disease survived the invasive infection. A positive outcome was also recorded for the single patient with IFD who had undergone allo-HSCT. Table 2 describes all IFD cases in this cohort, along with their clinical characteristics in detail.

Regarding IPA, the most prevalent clinical trait was fever, with an average temperature of 39.1 °C and an average duration of 17 days, despite broad-spectrum antimicrobials. IPA manifested with a dry cough in 62% of the patients. Regarding radiologic findings, the halo sign was evident in the chest’s high-resolution computed tomography (HRCT) in most cases (62%), while 25% of the patients presented with the air-crescent sign. Initial IPA treatment corresponded to a combination of intravenous LAMB and voriconazole in 7/8 patients (for an average of 19 days; range 11–31), succeeded by oral administration of voriconazole alone for an entire three-month course. Two IPA patients did not survive (2/7; 28.6%), but their death was attributed to refractory AML M5. One patient was treated successfully with isavuconazole for 48 days.

In fungemia cases, the only common clinical feature was fever (mean 38.8 °C; 38.1–39.6 °C), lasting 10 days (2–25). The central venous catheter (CVC) was removed in four out of the six respective cases. *Candida parapsilosis* was the most prevalent species to cause fungemia in this cohort (67%). *C. albicans* was identified in one case, while the case of *Exophiala dermatitidis* fungemia has been previously reported by our Department [14].

Non-invasive fungal infections affected 5.8% of the study population (9/154) and regarded 12 infections (half identified with *C. albicans* and half with *C. parapsilosis*). Most of these cases corresponded to nail infections (4/12), followed by rash located in the inner thighs, inguinal regions, and genitalia (two cases each). One case of *Candida* esophagitis was documented, while *C. parapsilosis* was isolated along with *Pseudomonas aeruginosa* in a patient with pus discharge from his gastrostomy. Most of the cases mentioned above were under prophylaxis (10/12).

As far as adverse events due to antifungals are concerned, 16% (4/25) of the patients administered with LAMB experienced allergic reactions, most commonly manifested with a skin rash developing within the first 15 min of the drug’s infusion (3/4) plus one patient who developed facial angioedema. Side effects were documented in 25% (2/8) of the patients who received voriconazole, which comprised visual hallucinations in one case and vomiting after co-administration with Sopa-K in the other.

## 4. Discussion

The available information for the incidence and outcome of IFDs in children is derived mainly from single-center retrospective studies [15]. In the present retrospective study, data were retrieved from the medical records of children and adolescents with neoplasia treated in the only Pediatric Hematology–Oncology Department in the island region of Crete during the last decade. Of interest, 8% of the treated patients suffered from one or more IFDs, and among them, most cases corresponded to possible/probable pulmonary aspergillosis (50%) followed by fungemia due to *Candida parapsilosis*. The mortality rate was as low as 12.5% (two fatal IFDs out of 16 episodes). These findings are in agreement with the published literature about prevalence (5–10%; range 2–15%, depending on geographic location, patient characteristics, and specific hospital practices) and mortality rates (ranging from 20 to 50%, depending on the IFD, the underlying neoplasia, and the prompt diagnosis and treatment) [16,17]. According to the literature, probable/proven IFDs’ prevalence among the pediatric AML population ranges between 5% and 15% (referring mostly to IPA followed by non-*albicans* invasive candidiasis) and is associated indirectly with fatal outcomes in up to 18% of patients [1,3,18,19]. AML diagnosis was strongly associated with IFD occurrence (OR 76.7; 95% CI: 16.6 to 353.1; *p* < 0.001) in this retrospective cohort study. Of note, 70% of AML patients developed an IFD, while only 2.4% of ALL cases (1/42; with *ETV6*::*RUNX1* fusion and under the high-risk protocol due to high measurable residual disease during induction) manifested an invasive fungal infection. The mean age of the children with IFD was 9.8 years, which agrees with other studies which associate the higher age of children with manifesting an IFD [20].

*C. parapsilosis* was the primary colonizer of mucus membranes and medical devices such as drainage catheters and CVCs, despite antifungal prophylaxis with micafungin in most cases. *C. parapsilosis* was also the predominant confirmed cause of an IFD under the same prophylaxis. As a result, antifungal prophylaxis did not have a statistically significant effect on IFD prevention. However, the population with IFD was small, comprising 12/154 children with different types of neoplasia. Hence, more studies are necessary to establish the definite role of antifungal prophylaxis in preventing IFDs. Considering the 6-year-old boy with B-ALL and IPA in our cohort, twice-a-week micafungin prophylaxis displayed a significantly lower occurrence of *Aspergillus* infections during the early phase of childhood ALL treatment, according to a recent study [21].

In agreement with the findings of the current study, the incidence of candidemia due to *C. parapsilosis* has increased in recent years, bearing clinical implications due to its decreased susceptibility to echinocandins [22]. However, no deaths were recorded due to fungemia or cellulitis caused by this yeast or combined with *C. paratropicalis* (as observed in this cohort’s case of cellulitis). All cases were successfully treated with a combination of LAMB and second-generation triazoles, followed by a step-down treatment with triazole only. Numerous studies are focusing on a personalized risk assessment for the decision on prophylactic or empirical antifungal administration [23]. The present study confirmed several risk factors that should trigger the investigations for an IFD: AML diagnosis, ICU setting for more than a week, administration of more than seven antimicrobials during the last trimester, history of severe neutropenia for 1.5 months, fungal colonization in sites other than the oral cavity, fever > 38.8 °C for >12 days, CRP levels above 10 mg/dL, treatment with prednisolone equivalent ≥ 0.3 mg/kg for more than three weeks, and IVIG administration. Intriguingly, IVIG administration often leads to false positive BDG levels that may remain detectable for more than two weeks. Therefore, BDG should not be used to diagnose an IFD within three weeks after IVIG administration [24]. Of course, medical devices colonized by fungi need immediate removal to prevent fungemia. The literature supports the equivalent prevalence of yeast and mold infections (50% and 50%), with this ratio remaining stable for both the 2018–2022 and 2013–2017 periods and despite the increased IFD incidence during the last 5 years [25]. An increase in bronchopulmonary mold infections and breakthrough IFDs has been noted by several studies in the field [5,26].

Broad-spectrum antibiotics, which are sometimes required but are primarily non-specific, are a highly relevant risk factor for developing systemic fungal infections [1]. A variety of antibiotic and antiviral regimens—more than seven in total number according to the present study—should be avoided. Moreover, all Pediatric Hematology–Oncology clinics should be regularly visited by an infectious disease specialist for more efficient use of antimicrobial substances. Emphasis should be placed on strict hand hygiene, safe catheter management, and catheter care in general [27]. Disinfection of the ward and medical equipment or objects that enter the patients’ wards with chlorine-based disinfectants can also eliminate *Candida* species that survive long on surfaces. The use of special air filters creates appropriate particle-free air flow and is crucial in the prevention of pulmonary IFDs [28]. Regarding antifungal therapy, seven out of eight cases of IPA received combined antifungal treatment with LAMB and voriconazole, although current guidelines based on randomized clinical trials promote monotherapy with voriconazole or isavuconazole. The combination of antifungals seems to represent a common practice in cases of severe and life-threatening diseases when management reasoning eclipses evidence-based medicine [29]. Of interest, the lower mortality rates of invasive mold infections reported in our study could be attributed to the early diagnosis and prompt administration of antifungal therapy. This is in line with previous work on the 2-fold increase in mortality following a ≥6 days delay in the administration of effective antifungal therapy [30].

The current study has several limitations that should be considered when interpreting the results, with its retrospective nature being the major one. The fact that all IPA cases were not proven or probable, the possibility of undiagnosed IFDs, and the presence of potential confounding variables limit the generalizability of the findings. In addition, the observational nature of the study precludes establishing causality between the identified factors and the outcomes.

## 5. Conclusions

The limited antifungal arsenal, combined with the well-documented resistance to the available agents, requires the development of alternative, effective, and safe strategies for IFD treatment. Moreover, prompt IFD diagnosis is crucial to prevent the observed high mortality rates, as late diagnosis equals a poor prognosis. Further studies, standardizing the existing technologies, and mastering novel tools (PCR and rapid point-of-care assays, T2Candida platform, etc.) are needed. IFDs are a significant burden among children with cancer, constituting an independent risk factor for both event-free and overall survival. Determining the relevant host factors and high-risk clinical traits could alleviate the issue of IFDs and breakthrough fungal infections.

## Figures and Tables

**Table 1:** Association of clinical and laboratory parameters with the development of IFD.

Parameter	IFD Group (N = 16)		No-IFD Group (N = 141)		*p* ^†^
	%	Mean (95% CI)	%	Mean (95% CI)	
ICU > 7 days	31.3%	7.9 (1–15)	4.3%	1.4 (0.3–2.4)	0.017
>7 antimicrobials	50%	7.3 (5–9.6)	15.6%	4.5 (3.9–5.1)	0.01
Neutropenia > 44 days	37.5%	44.6 (20.1–69.1)	17%	22.2 (17.5–26.9)	0.011
Fever > 38.8 °C	50%	38.9 (38.5–39.2)	29.1%	38.3 (38.1–38.4)	0.019
Fever > 12 days	43.8%	12.5 (4.7–20.3)	5.7%	4.1 (3.1–5.1)	<0.001
CRP > 10 mg/dL	37.5%	10.1 (6.6–13.7)	22%	5.9 (4.8–6.9)	0.011
Prednisolone equivalent ≥ 0.3 mg/kg for ≥3 weeks	31.3% ^‡^	26.6 (2.8–50.4)	21.3%	72.1 (60.3–83.8)	0.024
	%		%		*p*
Fungal colonization	31.3%		6.4%		0.007
IVIG	50%		17.9%		0.007
r/r disease	18.8%		10.6%		NS
Comorbidities	31.3%		22.9%		NS
HSCT	18.8%		8.5%		NS
Allo-HSCT	6.3%		2.2%		NS
Immunomodulatory agents	18.8%		19.1%		NS
GCSF	31.3%		33.3%		NS
Monocytopenia	87.5%		80.1%		NS
Lymphocytopenia	97.5%		84.2%		NS
Antifungal prophylaxis	87.5%		66%		NS

^†^ = corresponds to means; ^‡^ = during the last 60 days; NS = not significant.

**Table 2:** Clinical characteristics of patients with IFD.

Patient	Age (years)	Sex	Underlying Neoplasia	Treatment Protocol	IFD	Diagnostic Tools	Prophylaxis	Treatment	IFD Outcome
#1 Ep1	17	M	AML M1	AML–BFM 2019	*C. parapsilosis* & *C. paratropicalis* cellulitis	Biopsy + Culture	LAMB	LAMB–PCZ	Cured
#1 Ep2	17.5				IPA	HRCT + Clinical	LAMB	LAMB–VCZ	Cured
#1 Ep3	19			FLA–IDA	PJP	3X BAL–PCR + BDG	Pentamidine	SXT → PQ	Cured
#2 Ep1	12.5	M	AML M5	AML–BFM 2012	*C. parapsilosis* fungemia	Blood culture	MICA	LAMB–VCZ	Cured
#2 Ep2					IPA	HRCT + Clinical	MICA	LAMB–VCZ	Deceased
#3	3	M	LCA MB	COG–ACNS0334	*C. parapsilosis* fungemia	Blood culture	MICA	LAMB	Relapsed
#3 Rel						Blood culture		FCZ	Cured
#4	6	F	AML M5	AML–BFM 2012	IPA	HRCT + Clinical	MICA	VCZ–LAMB → CAS	Cured
#5	2	F	EPN	VEC	*C. albicans* fungemia	Blood culture	No	LAMB → MICA	Cured
#6	0.6	M	AML M7	AML–BFM 2019	*C. parapsilosis* fungemia	Blood culture	MICA	LAMB–VCZ	Cured
#7	8	F	AML M1	AML–BFM 2019	IPA	HRCT + Clinical	FCZ	LAMB–VCZ	Cured
#8	16	F	AML M5	AML–BFM 2019	IPA	HRCT + Clinical	CAS	LAMB–CAS	Deceased
#9	6	M	B–ALL	ALL IC–BFM 2009	IPA	HRCT + Clinical	LAMB	LAMB–VCZ → CAS	Cured
#10	12	F	HL	OΕΡA–COPDAC → IEP	IPA	HRCT + Clinical	No	LAMB–VCZ → CAS	Cured
#11	17	M	AML M3	AML–BFM 2019 HR + ATRA	IPA	BAL–PCR + Culture	MICA	ISZ	Cured
#12	4.5	M	EWS	EURO EWING 2012	*Exophiala dermatitidis* fungemia	Blood culture	VCZ	VCZ	Cured

ATRA = all-trans retinoic acid; BAL = bronchoalveolar lavage; BDG = (1–3)-β-D-Glucan; CAS = caspofungin; COPDAC = cyclophosphamide, vincristine, prednisolone, dacarbazine; EPN = ependymoma; F = Female; FCZ = fluconazole; FLA–IDA = fludarabine, cytarabine, idarubicin; IEP = ifosfamide, etoposide, prednisolone; ISZ = isavuconazole; LAMB = liposomal amphotericin B; LCA MB = Anaplastic/Large cell medulloblastoma; M = male; MICA = micafungin; OEPA = vincristine, etoposide, prednisolone, doxorubicin; PCR = polymerase chain reaction; PCZ = posaconazole; PQ = primaquine; SXT = trimethoprim/sulfamethoxazole; VCZ = voriconazole; VEC = vincristine, etoposide, cyclophosphamide.

## Data Availability

The data presented in this study are available on request from the corresponding author. The data are not publicly available due to privacy restrictions.
